# Beta-estradiol attenuates hypoxic pulmonary hypertension by stabilizing the expression of p27^kip1 ^in rats

**DOI:** 10.1186/1465-9921-11-182

**Published:** 2010-12-24

**Authors:** Dun-Quan Xu, Ying Luo, Yi Liu, Jing Wang, Bo Zhang, Min Xu, Yan-Xia Wang, Hai-Ying Dong, Ming-Qing Dong, Peng-Tao Zhao, Wen Niu, Man-Ling Liu, Yu-Qi Gao, Zhi-Chao Li

**Affiliations:** 1 Department of Pathology, Xijing Hospital, Fourth Military Medical University, Xi'an, 710032, PR China; 2 Department of Pathology and Pathophysiology, Fourth Military Medical University, Xi`an, 710032, PR China; 3 Key Laboratory of High Altitude Medicine, College of High Altitude Medicine Ministry of Education, Third Military Medical University, Chong Qing, 400038, PR China

## Abstract

**Background:**

Pulmonary vascular structure remodeling (PVSR) is a hallmark of pulmonary hypertension. P27^kip1^, one of critical cyclin-dependent kinase inhibitors, has been shown to mediate anti-proliferation effects on various vascular cells. Beta-estradiol (β-E2) has numerous biological protective effects including attenuation of hypoxic pulmonary hypertension (HPH). In the present study, we employed β-E2 to investigate the roles of p27^kip1 ^and its closely-related kinase (Skp-2) in the progression of PVSR and HPH.

**Methods:**

Sprague-Dawley rats treated with or without β-E2 were challenged by intermittent chronic hypoxia exposure for 4 weeks to establish hypoxic pulmonary hypertension models, which resemble moderate severity of hypoxia-induced PH in humans. Subsequently, hemodynamic and pulmonary pathomorphology data were gathered. Additionally, pulmonary artery smooth muscle cells (PASMCs) were cultured to determine the anti-proliferation effect of β-E2 under hypoxia exposure. Western blotting or reverse transcriptional polymerase chain reaction (RT-PCR) were adopted to test p27^kip1^, Skp-2 and Akt-P changes in rat lung tissue and cultured PASMCs.

**Results:**

Chronic hypoxia significantly increased right ventricular systolic pressures (RVSP), weight of right ventricle/left ventricle plus septum (RV/LV+S) ratio, medial width of pulmonary arterioles, accompanied with decreased expression of p27^kip1 ^in rats. Whereas, β-E2 treatment repressed the elevation of RVSP, RV/LV+S, attenuated the PVSR of pulmonary arterioles induced by chronic hypoxia, and stabilized the expression of p27^kip1^. Study also showed that β-E2 application suppressed the proliferation of PASMCs and elevated the expression of p27^kip1 ^under hypoxia exposure. In addition, experiments both *in vivo *and *in vitro *consistently indicated an escalation of Skp-2 and phosphorylated Akt under hypoxia condition. Besides, all these changes were alleviated in the presence of β-E2.

**Conclusions:**

Our results suggest that β-E2 can effectively attenuate PVSR and HPH. The underlying mechanism may partially be through the increased p27^kip1 ^by inhibiting Skp-2 through Akt signal pathway. Therefore, targeting up-regulation of p27^kip1 ^or down-regulation of Skp-2 might provide new strategies for treatment of HPH.

## Background

Pulmonary hypertension is a common complication of chronic hypoxic lung diseases, characterized by sustained elevation of pulmonary artery pressure and vascular resistance [1]. Pulmonary vascular structure remodeling (PVSR) is a hallmark of severe and advanced pulmonary hypertension, presenting several histological changes such as intima thickening, media hyperplasia and adventitia widening, peripheral vessels muscularization and vasoocclusive plexiform lesions [1,2]. Those changes finally lead to severe pulmonary hypertension, right ventricular hypertrophy and right heart failure, resulting in high morbidity and mortality [3]. Generally, there are 5 subsets of pulmonary hypertension classified according to the latest guidelines for the diagnosis and treatment of pulmonary hypertension [4]. Among these 5 subsets, pulmonary arterial hypertension (PAH), whose inducements including idiopathic, heritable, and connective tissue diseases, is predominantly suffered by women. According to epidemiological studies, there are about 2-3 times as many female as male patients [5-7]. The pathological lesions of PAH mainly affect the small pulmonary arteries (<500 μm of diameter), and are featured by medial hypertrophy, intimal proliferation and fibrotic changes, adventitial thickening, complex lesions, and thrombotic lesions [3,8]. Compared with PAH, the pathological changes of pulmonary hypertension due to lung diseases and/or hypoxia are characterized by medial hypertrophy and intimal obstructive proliferation of the distal pulmonary arteries [4,9]. In general, the severity of pulmonary hypertension due to lung diseases and/or hypoxia is usually from mild to moderate compared with PAH [4,10].

Despite the fact that PAH associated with idiopathic, heritable, and connective tissue diseases largely occurred to women, researchers reported that women with chronic obstructive pulmonary diseases exhibited a lower risk of mortality than men [11]. Consistent with this observation, earlier studies on HPH animal models have demonstrated that female rats developed less severe PH than male [12,13].

Current therapeutic strategies to pulmonary hypertension mainly include anticoagulation, prostacyclin, lung transplantation, atrial septostomy, and pulmonary endarterectomy [4,8]. New therapeutic strategies, such as prostacyclin analogues, endothelin-1 receptor antagonists, phosphodiesterase inhibitors and L-arginine, are emerging [14]. Furthermore, genetic therapy, stem cell therapy, and anti-proliferative therapies are also being under explored in laboratories [15].

Studies about estrogen have shown its various cardiovascular protective effects including prevention of coronary artery atherosclerosis [16], vasodilation of vessels [17], reduction of heart attacks [18] and attenuation of heart remodeling [19], etc. The molecular mechanisms of estrogen's cardiovascular protective effects involve decreasing induction of erythropoietin [20], inhibiting endothelin-1 expression [21], initiating nitride oxide synthesis [22], activating prostacyclin synthesis [23], and downregulating various adhension molecules [24]. Investigations also showed that estrogen can effectively relieve pulmonary hypertension of various etiologies including drugs, sclerosis, idiopathic, and other systematic diseases [25,26]. Additionally, Earley and Mukundan et al found estrogen markedly alleviated chronic hypoxia-induced pulmonary hypertension through modulating different molecules' expression [20,27]. Moreover, studies also revealed the anti-proliferation effects of estrogen in other proliferative vascular diseases [28,29].

Pulmonary vascular proliferation and remodeling are considered to be the central pathogenesis in the process of chronic hypoxia-induced pulmonary hypertension (HPH) [30]. In normal situation, most of the PASMCs in healthy adult are in a quiescent state [31], while proliferative PASMCs are found in the pulmonary hypertension arterioles which contribute to media thickening and vascular resistance [30,32]. Recent experimental studies indicate that cell cycle inhibition holds great potential as a therapeutic strategy for vascular proliferative diseases [33,34]. The cell cycle progression is regulated by kinds of cyclin-dependent kinases (CDKs) and their specific regulatory cyclins. The cyclin-dependent kinases inhibitors include INK4 family and cip family which can inhibit the activity of the CDK-cyclin complexes. The Cip/Kip family can inhibit all cyclin/CDK complexes *in vitro*, in which p27^kip1 ^is one of the core members controlling the cell cycle progression [35,36].

Though p27^kip1 ^was found years ago [37], it was recently recognized as an anti-oncogene protein [38,39]. Subsequent studies revealed p27 ^kip1 ^as an inhibitor of vascular smooth muscle cell, which might participate in vascular proliferative diseases [40]. Fouty and colleagues forward demonstrated that p27 ^kip1 ^is important in modulating PASMCs [41]. Moreover, Yu et al found that decreased functional p27^kip1 ^may contribute to PVSR associated with pulmonary hypertension, and up-regulated p27^kip1 ^mediate the inhibition effect on HPH [42].

The S phase kinase associated protein 2 (Skp-2) was found as a member of the large eukaryotic family of F-box proteins which functions specifically for the degradation of p27^kip1 ^[43]. Studies in these years demonstrated Skp-2 as a proto-oncogene and a positive regulator of cell cycle which involved in many diseases [44,45]. After activated by the phosphorylated Akt (Akt-P), Skp-2 mediates the proteolysis of p27^kip1 ^[46]. Now cancer therapeutic strategies targeting Skp-2 are becoming a hot topic [47,48].

In view of the mentioned above, we hypothesized that β-E2 may act on the Akt signal pathway and keep p27^kip1 ^from being degraded through down-regulation of Skp-2 under hypoxia condition. In this way, β-E2 may ameliorate chronic hypoxia-induced PVSR and pulmonary hypertension. Therefore, we adopted β-E2 to explore the roles of p27^kip1 ^and Skp-2 in the evolution of PVSR and HPH *in vivo*. Further, we verified their effects on cultured PASMCs *in vitro*.

## Methods

### Experimental groups

All animal experiments were approved by the Animal Care and Use Committee of the Fourth Military Medical University and complied with the Declaration of the National Institutes of Health Guide for Care and Use of Laboratory Animals (Publication No. 85-23, revised 1985). Male Sprague-Dawley rats (body weight 180-230 g) from the animal center of the Fourth Military Medical University (Xi'an, China) were used for all the experiments in our study.

Animals were randomly divided into 4 groups: 1) normoxia group, n = 7, 2) normoxia group treated with β-E2 (100 μg/kg via intraperitoneal injection), n = 7, 3) chronic hypoxia group, n = 7, and 4) chronic hypoxia group treated with β-E2 (100 μg/kg via intraperitoneal injection), n = 7. Animals designated for exposure to chronic hypoxia were housed intermittently in a hypobaric hypoxia chamber depressurized to 380 mmHg (oxygen concentration reduced to about 10%) and under hypoxia exposure for 10 h/d continuing 4 weeks. The normoxic control rats were housed at ambient barometric pressure (~718 mmHg, 21% oxygen). All animals were maintained in a 12:12-h light-dark cycle condition. The room temperature was air-conditioned at 25°C.

### Hemodynamic experiments and tissue preparation

After 4 weeks hypoxia exposure, the animals were anesthetized with 20% ethylurethanm (4 ml/kg i.p.), and a soft silicagel catheter linked to a Powerlab system (AD Instruments, Colorado Springs, CO, AUSTRALIA) was inserted into the right jugular vein. There were special pressure waveforms displayed on the monitor when the catheter arrived in the right ventricle chamber. The right ventricle peak systolic pressure (RVSP) was then recorded. Meanwhile, the mean carotid artery pressure (mCAP) was recorded via a special catheter inserted into the carotid artery.

After the hemodynamic data were recorded, sternotomy surgery was performed. Lungs together with heart were harvested and put in a culture plate with cold PBS. The weight of right ventricle (RV) and left ventricle plus septum (LV+S) were obtained, and the ratio of (RV/LV+S) was calculated as an index of RV hypertrophy. The lungs were dissected into 3-mm-thick slices at the same point (the lower lobe of the right lung) and placed in neutral buffer (pH 7.4) containing 10% formalin. The remained lungs were frozen in -80°C freezer for subsequent experiments.

### Morphological investigation

After soaked in 10% formalin for 72 hours, the slices were embedded in paraffin and sectioned into 4-μm-thick sections and hematoxylin and eosin staining was done. The stained lung sections were processed by a pathologist for light microscopic observation and photo images analysis. Pulmonary arteries, external diameter of which ranged from 50 to 200 μm, 5-6 vessels with approximate round shape were obtained from each individual animal, total 40 arteries were got from every group. The average size of the obtained vessels was 78 μm. The outside diameter and inside diameter of pulmonary arterioles were measured by an image-processing program (Image-Pro Plus, Version 5.1, Media Cybernetics, USA). The medial wall thickness, the cross sectional area of medial wall, and the total cross sectional vessel area were obtained. Pulmonary vascular structure remodeling was assessed by percent medial wall thickness (MT%) and percent medial wall area (MA%) two indices: (MT%) = 100 × (medial wall thickness)/(vessel semi-diameter); (MA%) = 100 × (cross-sectional medial wall area)/(total cross-sectional vessel area). All the morphological analysis was conducted in a double-blind method.

### Cell culture and treatment

Pulmonary artery smooth muscle cells (PASMCs) were obtained by tissue explant culturing method. Pulmonary arteries were isolated from adult male Sprague-Dawley rats as described above. After the adventitial layers together with surrounding tissue were removed, the pulmonary arteries were dissected into small pieces and placed in a culture flask. The flask was overturned placed and Dulbecco Eagle's minimum essential medium (DMEM) (HyClone, Logan, UT, USA) was added in. After 2-4 hours, the flask was carefully turned over, and the medium immersed the tissue pieces. The explanted tissue was cultured in DMEM supplemented with 100 U/ml penicillin, 0.1 mg/ml streptomycin, 2 mM L-glutamine, and 10% FBS and grown in humidified incubators (HH·CP-01W, Shanghai Boxun Industry & Commerce Co., Ltd. Medical Equipment Factory, Shanghai, China) at 37°C in 95% O_2 _, 5% CO_2_. The PASMCs grew out in about a week, and cell passage was performed when the cells grew to 70% confluence. Cells were used between passages 3 and 6. Smooth muscle cell identity was verified by positive staining for smooth muscle α-actin (mouse monoclonal antibody; Sigma, USA) at each passage (>95% of cells stained positive for smooth muscle α-actin). The cells were seeded at 1 × 10^7 ^cells per well in cell culture Petri dishes (JET BIOFIL inc, Canada) and allowed to grow for 2 days. Then the cells were undergone serum-starvation for 36 hours. The media was then changed to containing 5% FBS phenol-red-free DMEM (HyClone, Logan, UT, USA) with β-E2 in various dosages (10^-5^, 10^-7^, 10^-9 ^mol/L). There were total 5 groups for cell study, one normoxia group, and three hypoxia exposure groups treated with three different dosages of β-E2, and one under hypoxia exposure group alone. Cells were cultured either in 21% oxygen or 2% oxygen condition (HERAcell 240, Heraeus Inc, Germany) for another 48 hours. After treatment the cell lysates were obtained as described below for the Western blotting and RT-PCR analysis.

### Cell proliferation assay

To investigate the inhibition of β-E2 on hypoxia-induced proliferation of PASMCs, 3-(4,5-dimethylthiazal-2-yl)-2,5-diphenyltetrazoliumbromide (MTT) tests were completed. In a word, cells were seeded in 96-well cell culture Petri dishes at 4 × 10^4 ^cells per well according to the groups designated above and 3 dosages of β-E2 (10^-5^, 10^-7^, 10^-9 ^mol/L) was added in. After cultured for 48 hours under normoxia condition or hypoxia exposure, solution MTT was added into each well in a 5 mg/mL concentration. Cells were cultured for another 4 hours, and then dimethyl sulfoxide (DMSO) was added in. After vibrating for 10 minutes, the optical density values were detected at 490 nm wavelength by using a spectrophotometer (PowerWave XS, BioTek Inc, Vermont, USA).

### Western blotting analysis

Total lysates were obtained from harvested lung tissue and cultured PASMCs. Lung homogenates were prepared in RIPA lysis buffer, containing 50 mM Tris (pH 7.4), 150 mM NaCl, 1% Triton X-100, 1% sodium deoxycholate, 0.1% SDS, 2 mM NaF, 5 mM EDTA (pH 8.0), 1 mM sodium orthovanadate (Beyotime Inc, Jiangsu, China). The protease inhibitor of phenylmethylsulfonyl fluoride (PMSF, 1 mM) was added to the RIPA buffer in advance. Equivalent amounts of protein (30 μg) from each sample were separated on 12% SDS-polyacrylamide gels, and then transferred onto 0.22 μM nitrocellulose filter membranes (Millipore, Bedford, USA). The primary antibodies were p27^kip1 ^antibody (1:1000; Millipore, Bedford, USA), Skp-2 polyclonal antibody (1:50; Abcam, Cambridge, UK) and Akt-P (1:500; Cell Signaling Technology, Inc., Massachusetts, USA). The signals were detected by ECL kit (BestBio Inc, Shanghai, China).

### RNA extraction and reverse-transcription polymerase chain reaction (RT-PCR) investigation

Total RNA of lung tissue and cultured PASMCs were extracted by using Trizol agent (Invitrogen, Carlsbad, CA, USA). M-MLV reverse transcripase kits (BestBio Inc, Shanghai, China) were used to synthesize first-strand cDNA from 2.5 μg per sample of total RNA according to the manufacturer's instructions. The primer pairs were designed by primer premier 5 (PREMIER Biosoft International, Palo Alto CA, USA), and original information of cDNA were aligned in the GeneBank. The primers were checked and synthesized by Genescript Company (Nangjing, China). The primer pairs for p27^kip1 ^PCR (319 bp) were (forward chain) 5'-CTTGGAGAAGCACTGCCGAGAT-3' and (reverse chain) 5'-CCCTGGACACTGCTCCGCTA-3', for Skp-2 (396 bp) were (forward) 5'-TAAGCGTTAGGTCTTTGGAA-3' and (reverse) 5'-TGGTTGTGTGTGTCTGTGTC3', and for the housekeeping gene β-actin (270 bp) were (forward) 5'-ATCATGTTTGAGACCTTCAACA-3' and (reverse) 5'-CATCTCTTGCTCGAAGTCCA-3' respectively. PCR program for p27^kip1 ^was started by a 5 min denaturation procedure at 95°C, followed by 35 cycles of 95°C for 30 s, 51°C for 30 s and 72°C for 1 min, and a final extension at 72°C for 10 min; for Skp-2 was initiated by a 5 min denaturation step at 95°C, followed by 35 cycles of 95°C for 30 s, 59°C for 30 s and 72°C for 1 min, and a final extension at 72°C for 10 min; and for β-actin was began with a 5 min denaturation step at 95°C, followed by 35 cycles of 95°C for 30 s, 55°C for 30 s and 72°C for 1 min, and a final extension at 72°C for 10 min. After amplification was done, the products were separated by 1% agarose gel (containing 0.5 μg/ml ethidium bromide) electrophoresis. The gels were visualized by a gel visualizing system (BioSens SC 810, Shanghai Bio-Tech Inc, Shanghai, China) and densitometry was calculated using the imaging software (BioSens Digital Imaging 5, Shanghai Bio-Tech Inc, Shanghai, China).

### Statistical analyses

All values were expressed as mean ± SD. Statistical analysis was processed by using one-way ANOVA, followed by LSD test for post hoc multiple comparisons (SPSS for Windows version 16.0, Chicago, USA). Significant difference was accepted at *P *< 0.05.

## Results

### Beta-estradiol treatment attenuated chronic hypoxia-induced pulmonary artery remodeling and pulmonary hypertension in rats

The right ventricular systolic pressure (RVSP) was measured by catheterization via jugular vein to right ventricle, which substitutes for the pulmonary artery pressure. After 4 weeks hypoxia exposure, the RVSP of hypoxia group was significantly elevated compared with the normoxia group (47.36 ± 3.47 vs. 24.90 ± 1.66 mmHg; Table 1, n = 7, *P *< 0.01). In the hypoxia+β-E2 group, RVSP was significantly lower than the chronic hypoxia exposed group (35.17 ± 1.67 vs. 47.36 ± 3.47 mmHg; Table 1, n = 7, *P *< 0.01), even it was a little higher than those of normoxia group. There was no significant difference of the RVSP between the normoxia group and the normoxia treated with β-E2 group (24.90 ± 1.66 vs. 25.17 ± 1.20 mmHg; Table 1, n = 7, *P *< 0.01). There was no significant difference in the mCAP between each group (as shown in Table 1).

**Table 1:** Analysis of hemodynamic data and right ventricle hypertrophy index.

Group	mCAP (mmHg)	RVSP (mmHg)	(RV/LV+S)%
Normoxia	119.40 ± 8.90	24.90 ± 1.66	25.64 ± 2.19
Normoxia+β-E2	117.32 ± 6.02	25.17 ± 1.20	25.71 ± 1.89
Hypoxia	124.25 ± 3.31	47.36 ± 3.47*	39.01 ± 4.69*
Hypoxia+β-E2	119.39 ± 9.20	35.17 ± 1.67 ^#^	30.90 ± 1.47^#^

All values are expressed as mean ± SD (n = 7). *P < 0.01, significant difference from the normoxia group. ^#^*P *< 0.01, significant difference from the hypoxia group. mCAP: mean carotid artery pressure; RVSP: right ventricular systolic pressure; RV/LV+S: right ventricle/left ventricle + septum.

**Figure 1:**
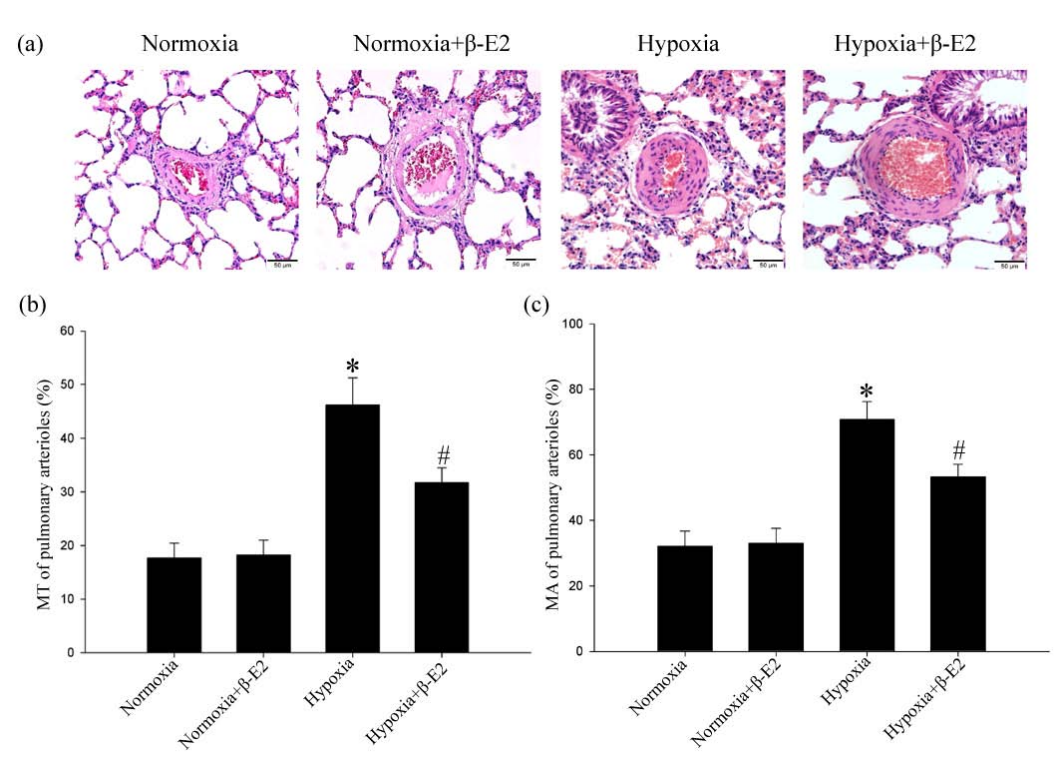
**Effects of β-E2 on chronic hypoxia-induced pulmonary vascular structure remodeling of rats**. (a) Hematoxylin and eosin staining of pulmonary arterioles (original magnification ×20). (b) Medial wall thickness (MT%) of pulmonary arterioles. (c) Medial wall area (MA%) of pulmonary arterioles. Scale bars = 50 μm. *P < 0.01, significant difference from the normoxia group. ^#^*P *< 0.01, significant difference from the hypoxia group. Arterioles of external diameter ranged from 50-200 μm, 5-6 vessels per animal, total 40 small arteries for each group, were obtained. Values are expressed as mean ± SD (n = 40).

Wall-thickened pulmonary arterioles with medial smooth muscle cell proliferation and hypertrophy, and inflammatory cell infiltration were observed in the chronically hypoxia-exposed rats lungs compared with those in the normoxia group. The adventitial thickening together with extracellular matrix accumulation, intima hyperplasia and endothelial cells proliferation were also observed in those rats' lungs under chronic hypoxia exposure. Destruction of lung alveolar structure can also be seen in the chronic hypoxia treated rats lungs. The vessel changes as described above in the hypoxia+β-E2 group were better than those of the hypoxia group (Figure 1a, Hematoxylin and eosin staining).

Furthermore, the medial wall thickness (MT)% of the arterioles, the index of pulmonary artery remodeling, was significantly elevated after chronic hypoxia exposure versus the normoxia group (Figure 1b, n = 40, *P *< 0.01). In the hypoxia+β-E2 group, (MT)% was notably smaller than in the hypoxia group (Figure 1b, n = 40, *P *< 0.01). There was no significant difference of the (MT)% between the normoxia group and normoxia+β-E2 group (Figure 1b). In accordance with the medial wall thickness, the medial wall area (MA)% in the hypoxia group was also significantly higher than in the normoxia group (Figure 1c, n = 40, *P *< 0.01). Consistent with the result of (MT)%, there was no significant difference of the (MA)% between the normoxia group and normoxia+β-E2 group (Figure 1c). Beta-estradiol treatment significantly decreased the (MA)% of the arterioles after hypoxia exposure compared with the hypoxia group (Figure 1c, n = 40, *P *< 0.01). Nevertheless, both (MT)% and (MA)% were still higher than that of the normoxia group (Figure 1b, c).

### Cell proliferation analysis of PASMCs

**Figure 2:**
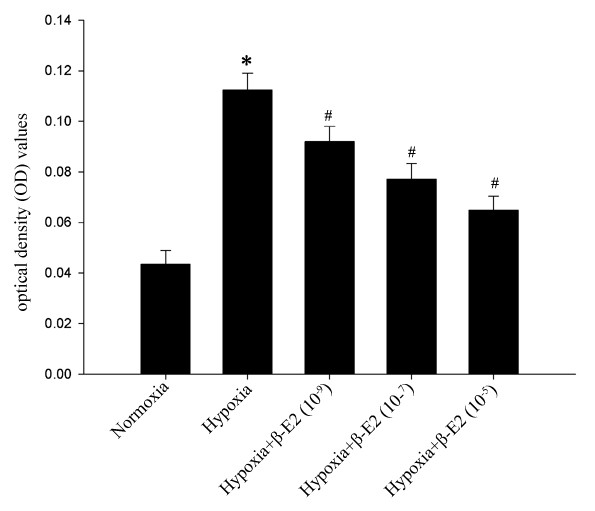
**Effects of β-E2 on rat PASMCs proliferation under hypoxia exposure (MTT assay)**. *P < 0.01, significant difference from the normoxia group. ^#^*P *< 0.01, significant difference from the hypoxia group. Values are expressed as mean ± SD (n = 3).

The OD values were representatives of the cells number. As shown in Figure 2, hypoxia exposure dramatically increased the OD values compared with those of the normoxia group (Figure 2, *P *< 0.01), that is, hypoxia exposure significantly increased the cell proliferation. The hypoxia-induced proliferation of PASMCs were obviously inhibited by three various dosages of β-E2 treatment (Figure 2, *P *< 0.01).

### Protein expression of p27^kip1 ^and Skp-2 in rat lung tissue and cultured PASMCs

**Figure 3:**
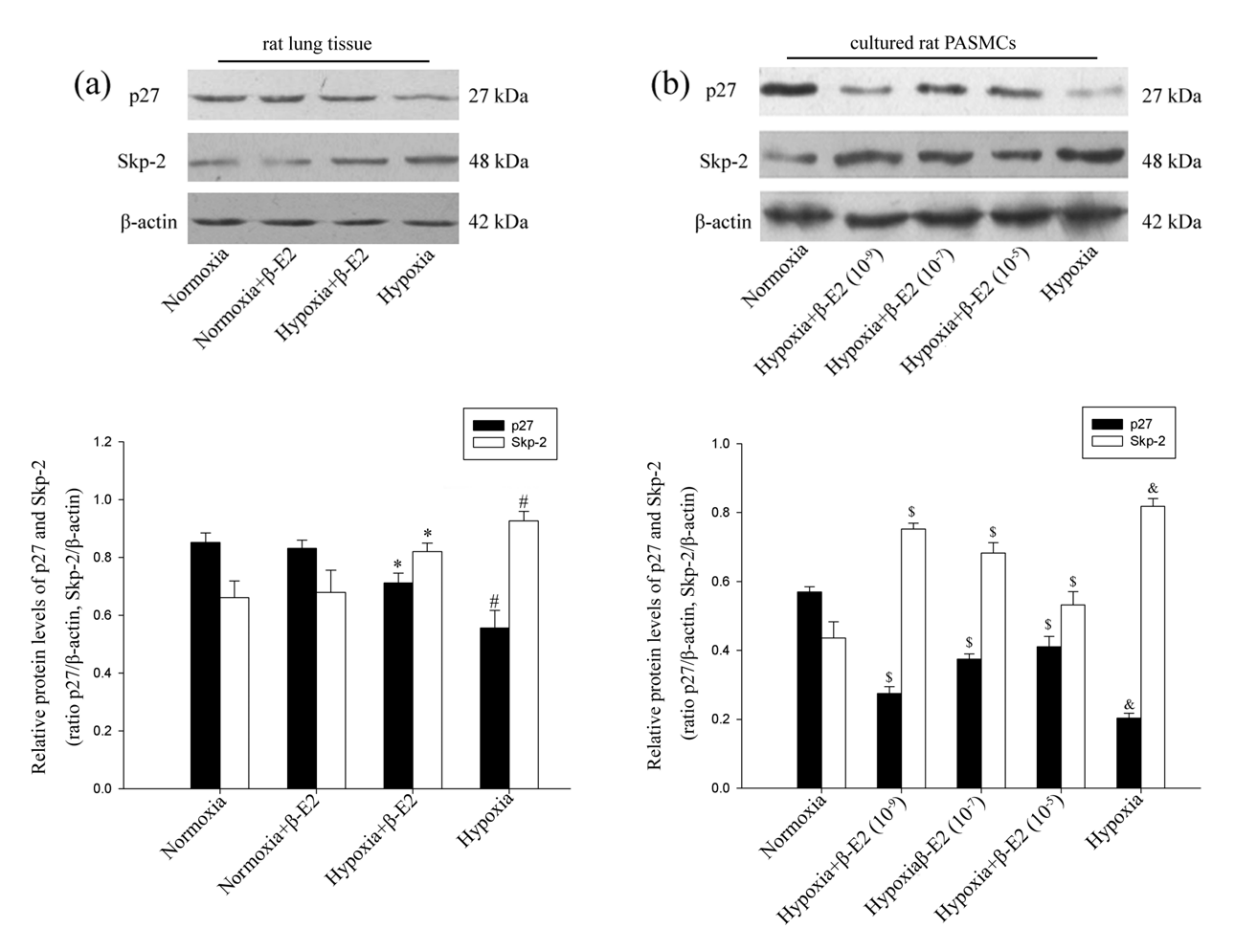
**Effects of β-E2 on p27 and Skp-2 expression in rat lungs and cultured rat PASMCs**. (a) Representative Western blotting analysis of p27 and Skp-2 protein levels in rat lungs. (b) Representative Western blotting analysis of p27 and Skp-2 protein levels in cultured rat PASMCs. *P < 0.05, significant difference from the hypoxia group. ^#^*P *< 0.05, significant difference from the normoxia group. ^$^*P *< 0.05, significant difference from the hypoxia group. ^&^*P *< 0.01, significant difference from the normoxia group. Values are expressed as mean ± SD (n = 3).

Aiming to know whether p27^kip1 ^and Skp-2 were involved in chronic hypoxia-induced pulmonary hypertension and artery remodeling, the protein levels of p27^kip1 ^and Skp-2 in the 4 experimental groups were compared. Western blotting results showed that the relative p27^kip1 ^level in hypoxia group was significant lower than that of the normoxia group. The relative p27^kip1 ^level in β-E2 treatment group was significantly higher than that of the hypoxia group (Figure 3a, *P *< 0.01). There was no notable difference of the relative p27^kip1 ^level between the normoxia and the normoxia+β-E2 group (Figure 3a). The results also showed that the relative Skp-2 level in hypoxia group was significantly higher than that of the normoxia group (Figure 3a, *P *< 0.01). The relative Skp-2 level in hypoxia+β-E2 group was significantly lower than that of the hypoxia group (Figure 3a, *P *< 0.01). In accordance with the results of p27^kip1^, there was no significant change of the Skp-2 relative level between the normoxia group and the normoxia+β-E2 (Figure 3a).

To further confirm if p27^kip1 ^and Skp-2 participated in the process of chronic hypoxia-induced pulmonary artery remodeling, the lysates of each group of PASMCs were used to do WB assays. As the results showed, compared with the normoxia group, the relative p27^kip1 ^levels was significant lower in hypoxia group. However, the relative p27^kip1 ^levels were markedly higher in all three dosages of β-E2 treatment groups than that of the hypoxia group (Figure 3b, *P *< 0.05). The relative p27^kip1 ^level in the β-E2 (10^-5 ^Mol/L) treated group was nearly 2 folds higher than in the hypoxia group (Figure 3b, *P *< 0.01). The data also revealed that hypoxia exposure notably escalated the expression of Skp-2 compared with the normoxia group (Figure 3b, *P *< 0.01). All three dosages of β-E2 treatment resulted in significant reduction of Skp-2 versus the hypoxia group (Figure 3b, *P *< 0.05).

### Protein expression of Akt-P in rat lung tissue and cultured PASMCs

**Figure 4:**
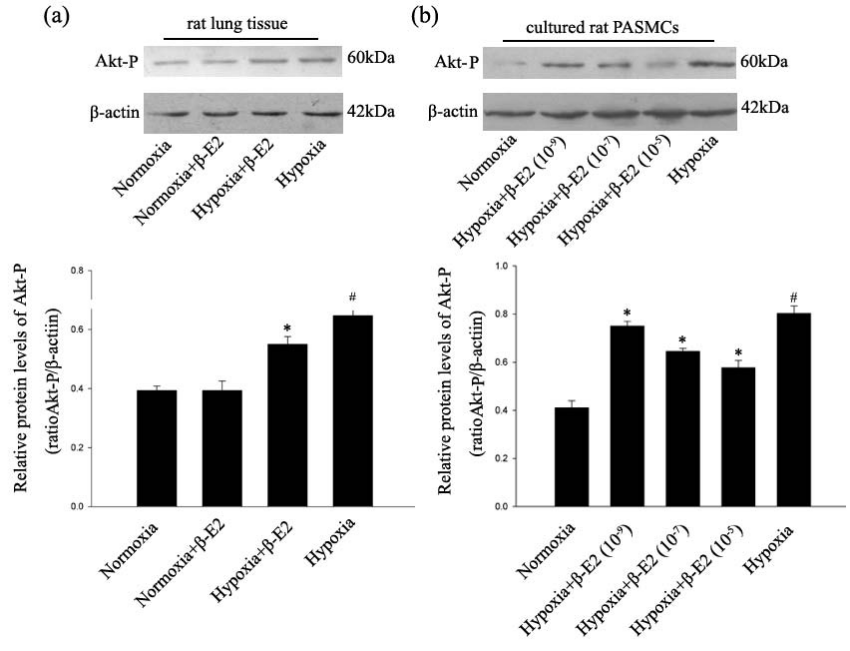
**Effects of β-E2 on Akt-P expression in rat lungs and cultured rat PASMCs**. (a) Representative Western blotting analysis of Akt-P protein levels in rat lungs. (b) Representative Western blotting analysis of Akt-P protein levels in cultured rat PASMCs. *P < 0.01, significant difference from the hypoxia group. ^#^*P *< 0.05, significant difference from the normoxia group. Values are expressed as mean ± SD (n = 3).

Our next step focused on phosphorylated Akt which initiates Skp-2 activation. Similar with Skp-2, the relative Akt-P level in hypoxia group was significantly higher than that of the normoxia group in rat lung tissue (Figure 4a, *P *< 0.01). The relative Akt-P level in hypoxia+β-E2 group was significantly lower than that of the hypoxia group (Figure 4a, *P *< 0.01).

In accordance with the expression of Skp-2, in cultured PASMCs, hypoxia exposure resulted in notably elevation of Akt-P versus the normoxia group (Figure 4b, *P *< 0.01). The expression of Akt-P in the three dosages of β-E2 treatment groups were all significantly lower compared with the hypoxia group (Figure 4b, *P *< 0.05).

### Changes in mRNA levels of p27^kip1 ^and Skp-2 in rat lung tissue and cultured PASMCs

**Figure 5:**
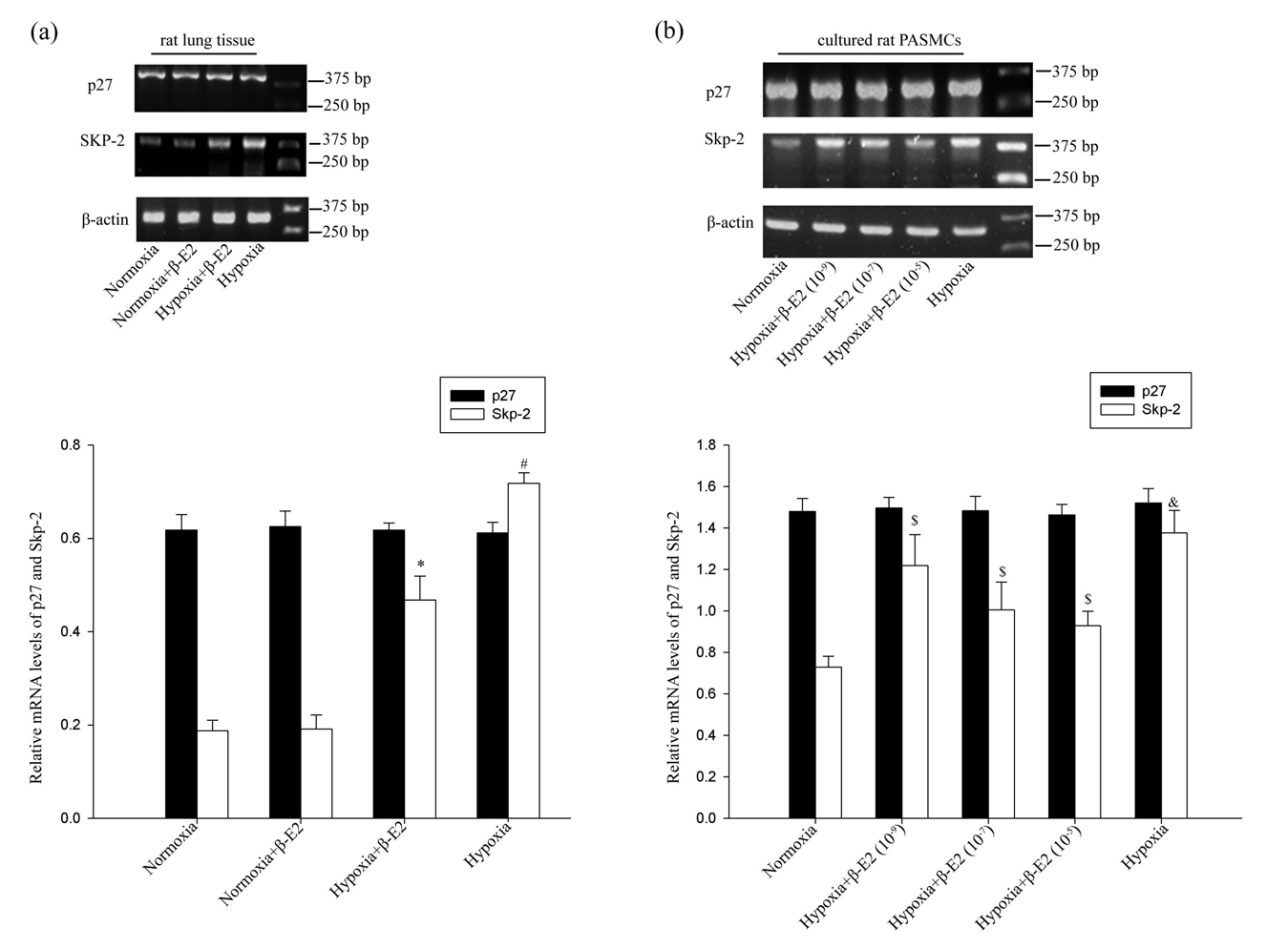
**Effects of β-E2 on mRNA expression in rat lungs and cultured PASMCs**. (a) Analysis of p27 and Skp-2 mRNA levels in rat lungs. (b) Analysis of p27 and Skp-2 relative mRNA levels in cultured rat PASMCs. *P < 0.01, significant difference from the hypoxia group. ^#^*P *< 0.01, significant difference from the normoxia group. ^$^*P *< 0.05, significant difference from the hypoxia group. ^&^*P *< 0.01, significant difference from the normoxia group. Values are expressed as mean ± SD (n = 3).

To further investigate whether p27^kip1 ^and Skp-2 were regulated at transcriptional level, the mRNA levels of p27^kip1^, Skp-2 and β-actin in lung tissue were analyzed by RT-PCR. The results of RT-PCR showed that there were no significant differences between all groups in relative p27^kip1 ^mRNA level, whether in rat lung tissue or in cultured PASMCs (Figure 5a, b).

On the other hand, the relative Skp-2 mRNA level in the hypoxia group was significantly elevated compared with that of the normoxia group in rat lung tissue (Figure 5a, *P *< 0.01). In the hypoxia+β-E2 group, the relative mRNA level of Skp-2 was significantly reduced compared with the hypoxia group (Figure 5a, *P *< 0.01). There was no significant difference between the relative Skp-2 level in rat lung tissue of normoxia group and normoxia+β-E2 (Figure 5a). In cultured PASMCs, the relative Skp-2 mRNA level was notably increased after hypoxia exposure compared with the normoxia group (Figure 5b, *P *< 0.01). All three different dosages of β-E2 treatment decreased the Skp-2 mRNA level in cultured PASMCs exposed to hypoxia (Figure 5b, *P *< 0.05).

**Figure 6:**
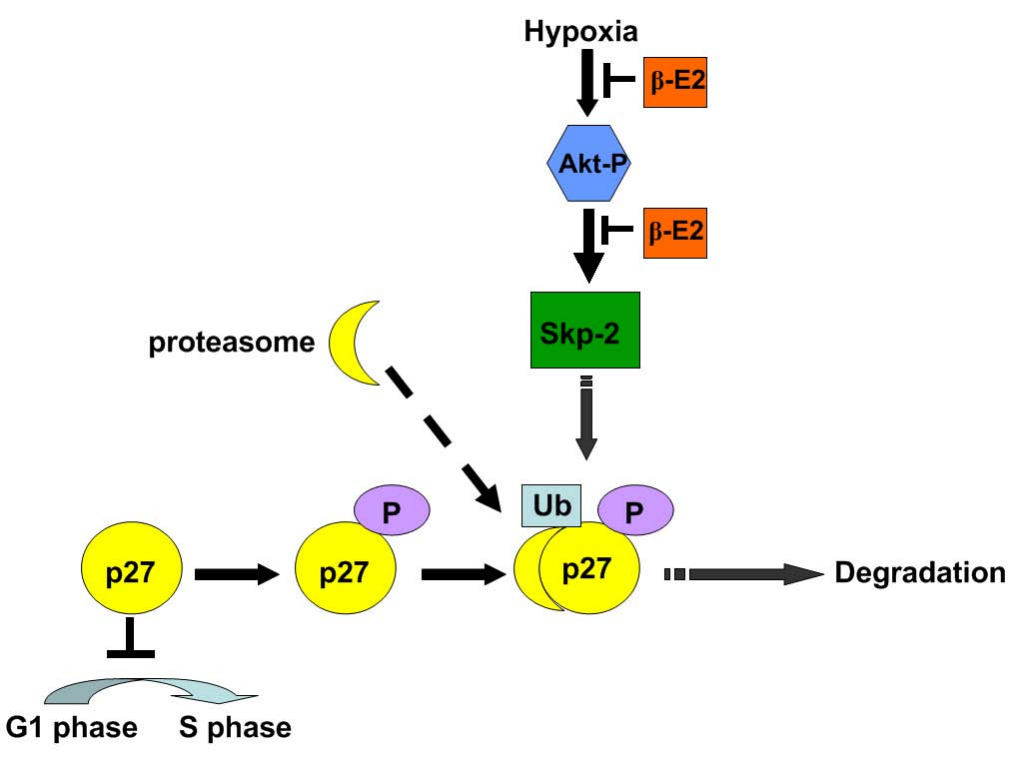
**Illustration of the potential mechanism of β-E2 effects on HPH **.

Taken together, these results suggested that hypoxia exposure resulted in PVSR and pulmonary hypertension. Beta-estradiol treatment exerted benefit effects on hypoxia-induced PVSR and pulmonary hypertension. The elevation of p27^kip1 ^and reduction of Skp-2 may play important roles in β-E2 attenuation effects on PVSR and HPH. The below diagram may elucidate the mechanism we explored in the present study (Figure 6).

## Discussion

Pulmonary hypertension is characterized by vasoconstriction and remodeling of small pulmonary arteries [3,8]. Media hyperplasia is the main pathologic change of the remodeling pulmonary arteries. The nature of the structural alteration and the mechanisms responsible for the pulmonary vasculature remodeling are complex and not yet fully elucidated [32]. Pulmonary hypertension accompanied with pulmonary vascular medial hyperplasia is mainly due to excessive PASMCs proliferation [30,32].

Charron and colleagues revealed cell cycle as a critical therapeutic target to prevent vascular diseases [34]. As an important CKI and anti-oncogene protein, p27^kip1^, is now being investigated for its inhibitory effects on cancer and vascular proliferative diseases [49,50]. Diez-Juan et al elucidated p27^kip1 ^functions as a suppressor in VSMCs proliferation, neovascularization, restenosis and atherosclerosis [51]. On the other hand, Skp-2 also draws lots of attentions from researchers for being an important proto-oncogene protein and the specific degradation ligase for p27^kip1^. Now, considerable studies focus on the therapeutic potential of p27 ^kip1 ^and Skp-2 on cancers or vascular diseases [47,52]. To date, there are few studies about the role of p27^kip1 ^on hypoxic pulmonary hypertension. More work need to be done to elucidate the beneficial effects of p27^kip1 ^on PVSR and HPH. As mentioned above, estrogen could effectively attenuate pulmonary hypertension and anti-proliferation in proliferative vascular diseases. Therefore, in this study we employed estrogen to explore the roles of p27 ^kip1 ^and Skp-2 in the evolution of PVSR and HPH.

It is interesting that p27^kip1 ^and Skp-2 indeed involved in the protective effects of β-E2 on the chronic hypoxia exposed rats. In the present study on the rat models, chronic hypoxia exposure resulted in significantly elevated RVSP, increased RV/LV+S, MT% and MA%, and marked media thickening of pulmonary arterioles. Western blotting data showed that hypoxia diminished the expression of p27^kip1 ^along with the escalation of Skp-2 and Akt-P. Beta-estradiol application reversed the reduction of p27^kip1^, elevation of Skp-2 and Akt-P, accompanied with the attenuation of pulmonary hypertension and PVSR induced by hypoxia. Consistent with the study *in vivo*, experiments *in vitro *also revealed the anti-proliferation effect of β-E2 on PASMCs. Moreover, the study *in vitro *also demonstrated that hypoxia resulted in marked reduction of p27^kip1 ^and augmentation of Skp-2 and Akt-P, which can be effectively reversed by β-E2 treatment.

As the WB data showed, β-E2 increased the expression of p27^kip1 ^protein in dosage-dependent manner. Moreover, Skp-2 and Akt-P decreased in direct proportion to the dosage of β-E2. On the other hand, RT-PCR data showed that p27^kip1 ^mRNA was not obviously changed by β-E2 application, neither *in vivo *nor *in vitro *experiments. The results are consistent with those reported by Hengst L [53]. In contrast with our results, Yu et al found that the p27^kip1 ^mRNA levels were significantly changed in hypoxia-exposed mice models compared with the heparin treated mice [42]. They also demonstrated in their study that p27^kip1 ^protein was up-regulated by heparin. In our opinion, different animal species and varied interventions in the researches may explain the discrepancy. Unlike p27^kip1^, Skp-2 was found to be both changed at mRNA and protein levels, indicating that β-E2 can decrease Skp-2 expression both through transcriptional and posttranscriptional mechanisms. In accordance with the study of Karin et al [46], the present study also demonstrated that the Akt signal pathway may account for the reduction of Skp-2.

Series of studies on estrogen testified that it could effectively alleviate various types of vascular diseases. However, there are many controversies over the protective effects of estrogen, especially over the anti-proliferation effect [54-61]. In this study, we found that β-E2 significantly inhibited PASMCs growth under hypoxia condition, both *in vivo *and *in vitro*. In our opinion, the different experimental conditions, different animal models and different treatments may explain the controversies.

The estradiol metabolite, 2-methoxyestradiol (2ME), was found able to abrogate injury-induced neointima formation to decrease proliferating SMCs and to up-regulate the expression of P27^kip1 ^[62]. Other researchers also revealed that 2ME can effectively attenuate bleomycin-induced pulmonary hypertension and fibrosis in rats [63]. Based on these studies, whether 2ME mediate the inhibition effect of β-E2 on PASMCs and elevation of P27^kip1 ^in chronic hypoxia-induced pulmonary hypertension remains to be determined.

Chronic hypoxia exposure increases intimal thickness of arterioles by causing hypertrophy and hyperplasia of the endothelial cells [30,64]. After being released from endothelium under chronic hypoxia condition, numbers of potent vasoactive substances then promote contraction and proliferation of PASMCs [65,66]. Investigations revealed that endothelial cells are closely associated with the PASMCs proliferation in the process of HPH. Though studies have shown the effects of estrogen on ECs including repressing adhesion molecules expression and enhancing expression of cytokines [27,67,68], more work should be done to explore whether estrogen could elevate the expression of P27^kip1 ^in endothelium and inhibit the proliferation of ECs under chronic hypoxia.

Taken together, our results demonstrated that β-E2 kept p27^kip1 ^from being degraded through decreasing the production of Skp-2. Subsequently, p27^kip1 ^prevented the chronic hypoxia induced PVSR and pulmonary hypertension through its inhibitory effects on PASMCs. Therefore, we concluded that stabilized p27^kip1 ^by β-E2 application indeed participated in the attenuation of PVSR and HPH. The down-regulated Skp-2 through Akt signal pathway may be responsible for the increased expression of p27^kip1^.

## Study limitations

(1) Though our data showed strong evidence that β-E2 could stabilize the expression of p27^kip1 ^and attenuate PVSR and HPH, whether it could have the same beneficial effects on humans with PAH remains unclear. (2) The gender differences were not studied, that is, whether β-E2 has the same protective effects on female rats as males needs to be explored. Since male rat models were employed in the study, whether male hormones involved in β-E2 attenuating HPH should also be furthered. (3) Though the study showed evidence of the inhibitory effects of β-E2 on PASMCs, it still seems somewhat insufficient for not considering the ECs.

## Conclusions

In brief, this study reveals that stabilized p27^kip1 ^may be a partial mechanism by which β-E2 exerts its protective effects on PVSR and HPH. Down-regulation of Skp-2 through Akt signal pathway may account for the increased expression of p27^kip1 ^after β-E2 application. So, up-regulating the production of p27^kip1 ^or down-regulating the expression of Skp-2 might be new strategies to treat PVSR and HPH.

## Abbreviations

Akt-P: phosphorylated Akt; β-E2: beta-estradiol; CDK: cyclin-dependent kinase; CKI: cyclin-dependent kinase inhibitor; ECs: endothelial cells; HPH: hypoxia-induced pulmonary hypertension; MA: medial wall area; mCAP: mean carotid artery pressure; 2ME: 2-Methoxyestradiol; MT: medial wall thickness; PASMC: pulmonary artery smooth muscle cell; PVSR: pulmonary vascular structure remodeling; RV/LV+S: right ventricle/left ventricle+septum; RVSP: right ventricular systolic pressure; Skp-2: S phase kinase associated protein 2

## Competing interests

The authors declare that they have no competing interests.

## Authors' contributions

DQX, YL and Yi L designed the experiment, carried out the data analysis and drafted the manuscript. JW reviewed the manuscript for the first time. DQX, JW and BZ carried out the animal experiment. MX and YXW did the histopathological analysis and cultured the PASMCs. MX and HYD carried out the RT-PCR and Western blotting assays. MQD, PTZ, WN and MLL participated in directing the experimental techniques and coordination of the studies. YQG and ZCL conceived the total study and critically reviewed the manuscript. All authors have read and approved the final manuscript.
